# Case Report: Myelodysplastic/myeloproliferative neoplasm with concurrent SF3B1, ASXL1, JAK2 and CBL mutations and <15% bone marrow ringed sideroblasts

**DOI:** 10.3389/fonc.2025.1622820

**Published:** 2025-07-23

**Authors:** Yifan Wang, Shengyu Jin

**Affiliations:** ^1^ Yanbian University, Yanji, Jilin, China; ^2^ Department of Hematology, Yanbian University Hospital, Yanji, Jilin, China

**Keywords:** myelodysplastic/myeloproliferative neoplasm (MDS/MPN), SF3B1 mutation, JAK2 mutation, <15% bone marrow ringed sideroblasts, WHO classification criteria

## Abstract

This first-reported case of SF3B1-mutated myelodysplastic/myeloproliferative neoplasm with thrombocytosis (MDS/MPN-SF3B1-T), harboring coexisting ASXL1, JAK2 p.R683G, and CBL mutations challenges conventional genomic prognostic paradigms. A 72-year-old woman presented with anemia (Hb 91 g/L), thrombocytosis (Platelets 502×10^9^/L), and 10% bone marrow ring sideroblasts, fulfilling 2022 WHO diagnostic criteria through molecular precedence of SF3B1 p.K700E (VAF 40.5%) despite subthreshold sideroblasts. Comprehensive genomic profiling revealed a unique quadruple mutation signature: ASXL1 p.G646Wfs*12 (9.8% VAF), JAK2 p.R683G (17.5%), and CBL p.R149Q (16.2%), with preserved karyotype. Functional analyses demonstrated mutation-specific pathobiological crosstalk: 1) SF3B1-mediated mitochondrial iron mislocalization (ALAS2 splicing defects, ABCB7 downregulation) synergized with ASXL1-driven epigenetic repression of erythroid transcription factors (GATA1, KLF1), exacerbating anemia; 2) JAK2 p.R683G’s partial kinase activation combined with CBL-dependent RAS/MAPK signaling sustained thrombocytosis through megakaryocytic hyperplasia. Despite harboring high-risk ASXL1 truncation, the patient maintained hematologic stability for six months without therapy, exhibiting declining platelet counts and improving Hb. This apparent genotype-phenotype discordance was attributed to clonal equilibrium (SF3B1 dominance suppressing ASXL1 leukemogenicity) and mutation-specific signaling attenuation (JAK2 R683G’s suboptimal kinase activation). Our findings necessitate revision of therapeutic algorithms for molecularly complex, treatment-naive elderly patients, particularly in resource-limited settings where socioeconomic factors critically influence management strategies.

## 1 Introduction

The 2022 WHO classification redefines SF3B1-mutated MDS/MPN with thrombocytosis (MDS/MPN-SF3B1-T) by prioritizing molecular over morphological criteria, yet real-world validation in molecularly complex cases remains limited. Co-occurring mutations, particularly ASXL1 truncations and JAK2 variants, may alter disease biology in elderly patients, where clonal heterogeneity and socioeconomic factors complicate management.

We report the first case of MDS/MPN-SF3B1-T harboring concurrent SF3B1 p.K700E, ASXL1 p.G646Wfs*12, JAK2 p.R683G, and CBL p.R149Q mutations. Despite <15% ring sideroblasts (RS) and high-risk mutations, the 72-year-old patient maintained stable hematopoiesis for six months without therapy, underscoring the need for nuanced refinement of conventional prognostic models in genomically complex elderly patients, where IPSS-M/R low-risk stratification accurately predicts indolent progression but fails to integrate mutation-specific clonal competition dynamics. Our findings validate molecular-driven classification while exposing critical gaps in therapeutic algorithms for elderly patients with genomic complexity. The case underscores the necessity of age-adapted management frameworks integrating clonal dynamics and resource constraints.

## 2 Case report

### 2.1 Case presentation

A 72-year-old female patient presented to Yanbian Hospital on 23 July 2024, with a chief complaint of progressive fatigue. Physical examination revealed: temperature 36.2°C, pulse 72 beats/min, respiratory rate 18 breaths/min, blood pressure 115/63 mmHg. No pallor, jaundice, skin rash, or petechiae were observed. Superficial lymph nodes were non-palpable, and there was no sternal tenderness. Clear breath sounds were noted bilaterally without crackles or wheezes. The abdomen was soft with normal bowel sounds, no tenderness or rebound pain, and no hepatosplenomegaly. Costovertebral angle tenderness and lower extremity edema were absent.

### 2.2 Investigations

**Figure 1:**
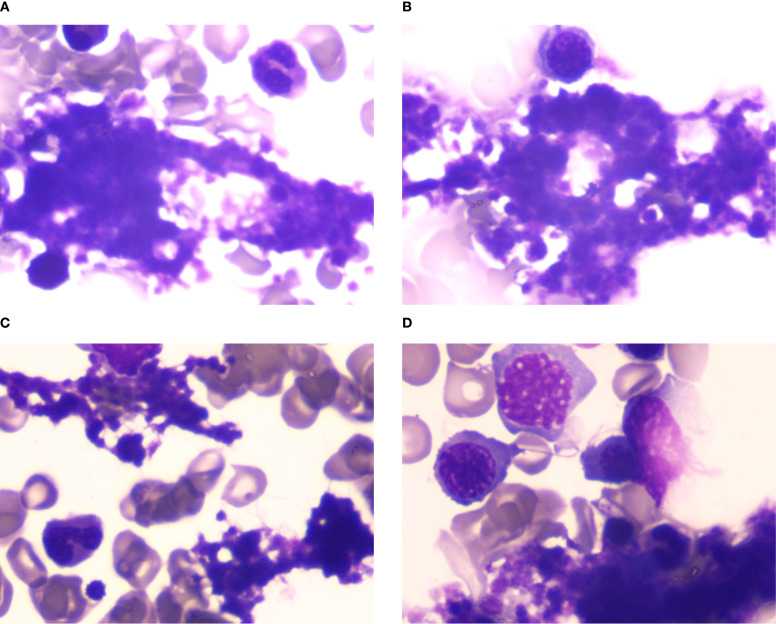
**(A–D)** Bone marrow cytomorphology analysis. Bone marrow aspirate morphology demonstrated a moderately hypercellular marrow with notable megakaryocytic hyperplasia and platelet aggregates, consistent with thrombocytosis.

**Figure 2:**
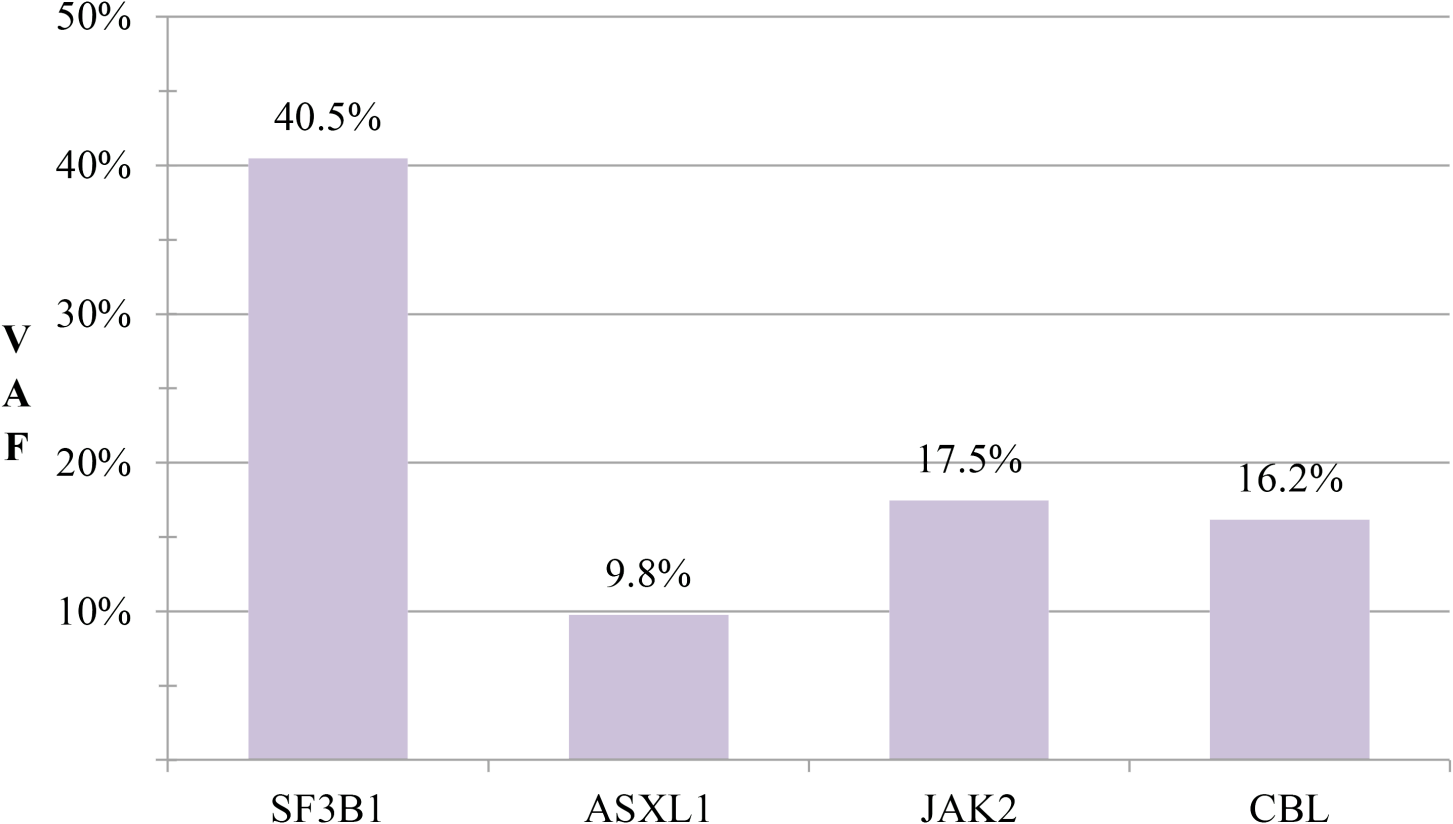
Mutation profile comparison. Comparative analysis of variant allele frequencies (VAFs) for SF3B1, ASXL1, JAK2, and CBL mutations.

Laboratory findings: Complete blood count: White blood cells: 7.54×10^9^/L, Red blood cells: 3.18×10^12^/L, Hemoglobin: 91 g/L, Platelets: 502×10^9^/L. Anemia panel: Ferritin: 317.40 ng/mL, Erythropoietin: 32.27 mIU/mL. Bone marrow studies: Aspirate morphology (**Figures 1A–D**): Cellularity: Normocellular (granulocytic series 47.5%, erythroid series 37.5%, myeloid-to-erythroid ratio 1.27:1). Granulopoiesis: Decreased proportions of metamyelocytes and band forms, with relative neutrophilic hyperplasia; eosinophils present. Erythropoiesis: Erythroid hyperplasia with increased late-stage erythroblasts; anisocytosis observed. Megakaryocytes: 3 megakaryocytes per slide with prominent platelet clustering. Iron staining: Ring sideroblasts (10% of erythroid precursors), bone marrow iron stores (+). Biopsy and immunohistochemistry (**Supplementary Figures S1A–D**): Reticulin staining: Grade MF-2 fibrosis. Flow cytometry analysis (**Supplementary Figure S2**): 0.12% of nucleated cells, no immunophenotypic aberrancy. Cytogenetic and molecular studies: Karyotype (**Supplementary Figures S3A, B**): 46,XX[20] (normal female karyotype). Next-generation sequencing (**Supplementary Tables S1**–**S3**): SF3B1 p.K700E (VAF 40.5%), ASXL1 p.G646Wfs*12 (VAF 9.8%), JAK2 p.R683G (VAF 17.5%), CBL p.R149Q (VAF 16.2%) (**Figure 2**).

### 2.3 Comprehensive assessment

The treatment strategy was co-developed through a clinician-patient partnership, incorporating:

#### 2.3.1 Diagnostic and therapeutic challenges in a resource-limited setting

The patient, a 72-year-old woman from a rural farming community in northeastern China, initially sought medical attention for progressive fatigue but was reluctant to undergo further diagnostic workup, including bone marrow examination, due to financial constraints and limited understanding of her condition. However, given her unexplained anemia (Hb 91 g/L) and marked thrombocytosis (502 × 10^9^/L), our team strongly recommended a comprehensive diagnostic evaluation, including morphology, immunophenotyping, cytogenetics, and molecular profiling (MICM), to establish an accurate diagnosis. We emphasized that empirical treatment without definitive diagnosis could obscure future bone marrow findings, complicating subsequent management. After detailed counseling, the patient and her family consented to proceed with the recommended investigations.

#### 2.3.2 Socioeconomic barriers to treatment and ethical consideration

Following diagnosis, the proposed treatment regimen (e.g., azacitidine, luspatercept) posed significant financial burden, leading the patient and her family to request deferred intervention.

After thorough assessment of: Patient-specific factors (ECOG 1, stable hematologic parameters, no high-risk cytogenetics). Financial toxicity concerns (limited household income, lack of insurance coverage for novel therapies). Molecular risk-benefit analysis (SF3B1-dominant clone mitigating ASXL1-associated high-risk features). we adopted an active surveillance strategy, prioritizing affordability while maintaining safety. Given the patient’s remote residence and inability to travel frequently, we optimized monitoring through: Monthly CBC monitoring via local clinics to detect disease progression. Emergency financial assistance pathways. Deferred therapy unless clinical progression, with clear escalation thresholds to trigger reevaluation.

#### 2.3.3 Alignment with clinical guidelines in resource-limited settings

While WHO’s WHA71.8 resolution (1) advocates equitable access to essential medicines, its direct applicability to this case is limited, as the patient’s primary barrier was affordability of targeted therapies rather than systemic drug shortages. Instead, our approach aligns with NCCN Guidelines^®^ for lower-risk MDS/MPN, which endorse observation in asymptomatic patients with stable disease, particularly when treatment costs outweigh proven survival benefits in resource-limited settings (2). This pragmatic strategy balances diagnostic rigor, ethical autonomy, and socioeconomic realities, ensuring patient-centered care without compromising long-term outcomes.

### 2.4 Outcome and follow-up

**Figure 3:**
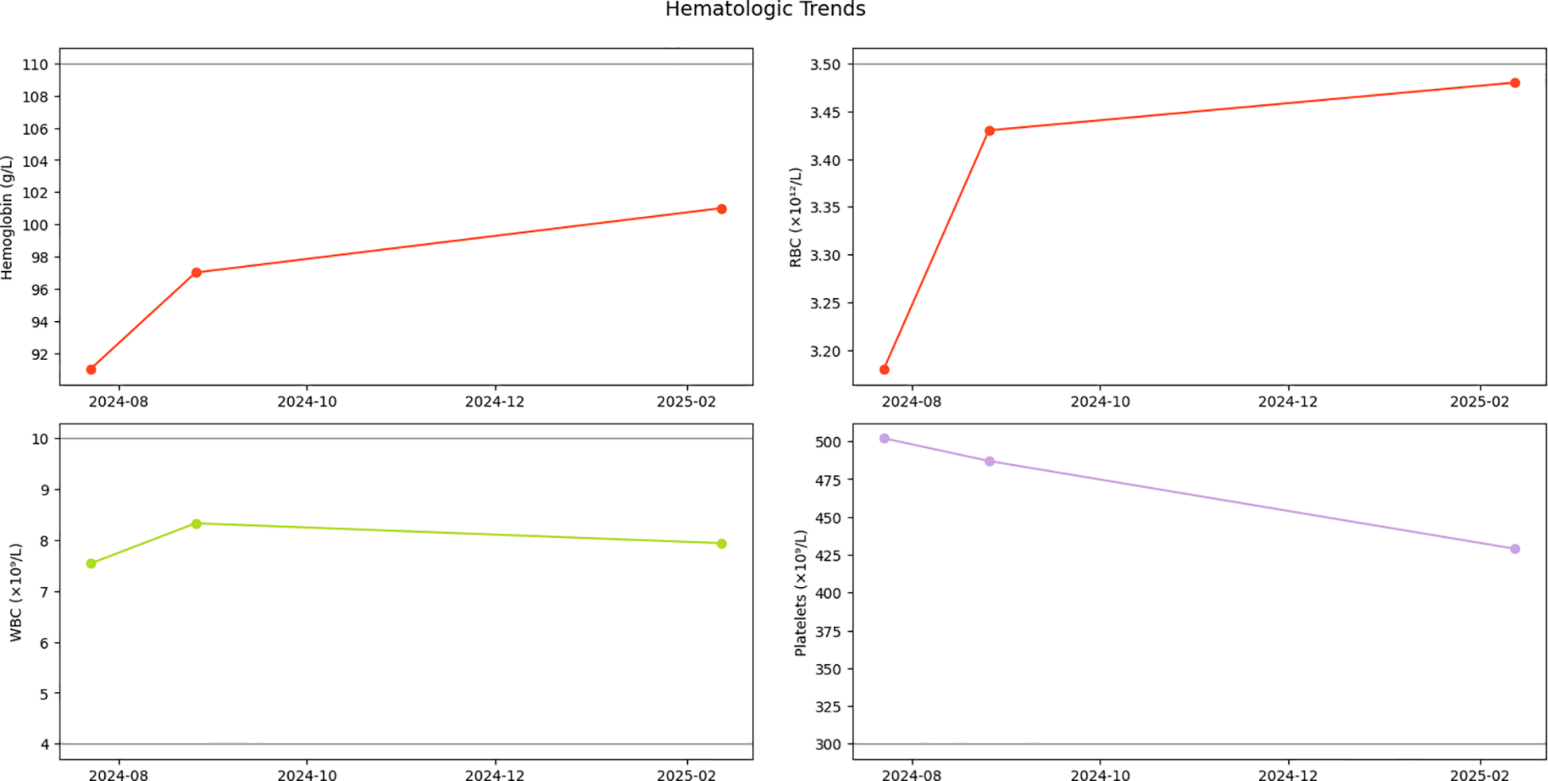
Hematologic parameter trends. Longitudinal trajectory of hemoglobin (Hb), red blood cells (RBCs), white blood cells (WBCs), and platelet counts during initial diagnosis and follow-up monitoring. Gray dashed lines indicate reference thresholds for normal ranges.

Serial monitoring revealed dynamic yet stable hematologic parameters during the six-month observational period. At initial follow-up on 26 August 2024, complete blood count demonstrated: white blood cells 8.33×10^9^/L, red blood cells 3.43×10¹²/L, hemoglobin 97 g/L, and platelets 487×10^9^/L. Notably, platelet counts showed a gradual descending trend without therapeutic intervention. Subsequent evaluations demonstrated sustained stability, with the latest results on 12 February 2025 documenting: white blood cells 7.94×10^9^/L, red blood cells 3.48×10¹²/L, hemoglobin 101 g/L, and platelets 429×10^9^/L (**Figure 3**). Importantly, no disease progression signals emerged during surveillance, consistent with the adopted watchful waiting strategy.

## 3 Discussion

### 3.1 Diagnostic evolution and molecular stratification

The diagnosis of MDS/MPN-SF3B1-T in this case exemplifies the critical evolution of WHO 2022 classification criteria.

#### 3.1.1 Historical context

The 2016 WHO classification mandated ≥15% ring sideroblasts for MDS/MPN-RS-T diagnosis, regardless of SF3B1 status (3). This morphological threshold missed 23% of SF3B1-mutated cases with <15% RS but similar clinical course (4).

#### 3.1.2 2022 WHO revisions

Two evidence-based changes redefine diagnostic standards: Molecular primacy: SF3B1 mutation alone establishes diagnosis (RS threshold eliminated) (5). Nomenclature shift: “SF3B1-T” replaces “RS-T” to reflect molecular pathogenesis. Our patient’s presentation (10% RS, SF3B1 VAF 40.5%) aligns perfectly with this framework.

#### 3.1.3 Exclusion criteria nuance

While JAK2 V617F excludes MDS/MPN-SF3B1-T diagnosis (5, 6), our patient’s rare R683G variant, lacks canonical MPN-driving potency, maintains diagnostic eligibility per 2022 WHO technical notes (5).

#### 3.1.4 Differential diagnosis

The diagnosis of MDS/MPN-SF3B1-T in this case required rigorous exclusion of overlapping entities through integrated clinicopathologic and molecular assessment:

Chronic Myelomonocytic Leukemia (CMML): Peripheral blood monocyte count remained persistently <1×10^9^/L. Absence of dysgranulopoiesis in bone marrow morphology, contrasting with CMML’s characteristic granulocytic dysplasia. Molecular exclusion: No mutations in SRSF2 or RAS pathway genes, which occur in >80% of CMML cases.

Other MDS/MPN Subtypes: Atypical CML (aCML): Ruled out by lack of prominent granulocytic hyperplasia (myeloid:erythroid ratio 1.27:1) and absence of CSF3R or SETBP1 mutations. MDS/MPN-U: Excluded by meeting definitive SF3B1-T criteria through the molecular precedence.

Essential Thrombocythemia (ET): Iron-stainable marrow iron stores and ring sideroblasts (10%) contradicted ET’s typical iron depletion. JAK2 p.R683G’s attenuated signaling precluded classical ET phenotype.

This systematic exclusion approach reinforces diagnostic specificity, particularly given the patient’s thrombocytosis and borderline ring sideroblast percentage. The 2022 WHO classification’s prioritization of SF3B1 mutation—even with subthreshold sideroblasts—was pivotal in resolving diagnostic uncertainty, while its emphasis on mutation combinations allowed for nuanced risk stratification despite phenotypic divergence from classic SF3B1-mutant disease. This integrative molecular-morphological framework enables precise categorization of biologically heterogeneous cases, overcoming limitations of prior morphology-centric systems.

### 3.2 Molecular pathogenesis and signaling pathway alterations

The patient’s unique quadruple mutation profile (SF3B1, ASXL1, JAK2, and CBL) demonstrates complex interactions between splicing dysfunction, epigenetic regulation, and proliferative signaling. We analyze each mutation’s mechanistic contribution with supporting evidence from recent studies (**Table 1**).

**Table 1:** Functional, prognostic, and therapeutic profiles of key mutations in MDS/MPN-SF3B1-T.

Mutation	Key functional impact	Prognosis	Therapy relevance
SF3B1 p.K700E	Splicing defects → mitochondrial iron overload, impaired erythropoiesis.	Favorable (if dominant).	Luspatercept-responsive.
ASXLl p.G646Wfs*12	PRC2 disruption → epigenetic dysregulation, erythroid suppression.	High-risk (AML progression).	Azacitidine/venetoclax-sensitive.
JAK2 p.R683G	Partial kinase activation → thrombocytosis (low STATS signaling).	Lower thrombotic risk.	Moderate JAK inhibitor response.
CBL p.Rl49Q	Impaired RTK degradation → sustained RAS/MAPK; paradoxically destabilizes JAK2.	Context-dependent (may limit growth).	JAK/MEK dual inhibition potential.

#### 3.2.1 SF3B1 p.K700E: splicing dysregulation in ineffective erythropoiesis

The SF3B1 p.K700E mutation (VAF 40.5%), a hotspot variant disrupting spliceosome fidelity, drives erythroid failure. SF3B1 p.K700E induces aberrant splicing of iron metabolism genes. ABCB7 intron retention reduces mitochondrial iron exporter levels, promoting mitochondrial iron overload (7, 8). ALAS2 exon skipping impairs heme synthesis, uncoupling iron accumulation from hemoglobin production (7). Mispackaged pre-mRNAs trigger nuclear retention, depleting erythroid transcription factors (GATA1, KLF1) critical for differentiation (9).

SF3B1 mutations induce molecular iron mislocalization before morphological RS manifestation, justifying WHO 2022’s elimination of the 15% RS threshold (8). Dominant clonal status (VAF 40.5%) predicts superior response to luspatercept via SMAD2/3 pathway modulation (10).

This molecular hierarchy confirms SF3B1 mutations as both diagnostic biomarkers and therapeutic targets, independent of traditional morphological criteria.

#### 3.2.2 ASXL1 p.G646Wfs*12: epigenetic deregulation and clinical implications

The ASXL1 truncation drives myeloid neoplasms through PRC2-mediated epigenetic dysregulation and confers distinct therapeutic vulnerabilities.

PRC2 Disruption and Transcriptional Derepression

ASXL1 truncations destabilize the PRC2 complex causing global H3K27me3 loss at polycomb targets (e.g., HOXA9), activating leukemogenic pathways. Collapse of H3K4me2/H3K27me3 bivalent domains at differentiation promoters, priming stemness genes (11). ASXL1 mutations occurred more frequently in MDS/MPN overlap syndromes than classical MDS (12–14), retaining independent adverse prognosis with shorter OS and higher leukemic transformation risk (6, 13). This aligns with its incorporation in the IPSS-M model for cross-subtype risk stratification, validated in multi-institutional cohorts (4, 15). The shared prognostic impact underscores ASXL1’s role as a pan-myeloid driver of aggressive disease biology.

Mutation-Specific Therapeutic Vulnerabilities

Azacitidine Sensitivity: ASXL1 truncation increases BIM expression and enhances azacitidine-induced apoptosis, potentially through epigenetic derepression. Direct evidence of BIM promoter demethylation requires further validation. Venetoclax-Hypomethylating Agent (HMA) Synergy: The ASXL1 mutation disrupts H3K27me3-mediated epigenetic repression, leading to MCL-1 upregulation and consequent cancer cell dependence on MCL-1 for survival, which confers resistance to BCL-2-specific Venetoclax; hypomethylating agents (e.g., azacitidine) transiently suppress MCL-1 expression, forcing malignant cells to shift their survival dependency to BCL-2, thereby enabling Venetoclax to effectively trigger apoptosis through BCL-2 inhibition—this dual mechanism of “HMA-mediated MCL-1 suppression plus Venetoclax-induced BCL-2 blockade” synergistically overcomes the epigenetic resistance of ASXL1-mutated cells (16).

#### 3.2.3 JAK2 p.R683G: structural perturbation and signaling attenuation

The JAK2 p.R683G mutation in the pseudokinase domain (JH2) exhibits distinct molecular characteristics compared to the canonical V617F variant, as evidenced by structural and functional studies:

JH2 Domain Destabilization

The R683G substitution destabilizes the JH2 pseudokinase domain by disrupting key hydrogen bonds within its inter-lobe linker region, as evidenced by molecular dynamics simulations showing a partially unfolded conformation (RMSD=2.3 Å versus wild-type). This structural perturbation significantly compromises JH2’s autoinhibitory capacity to constrain the JH1 kinase domain, while maintaining an intermediate activation state that permits constitutive signaling without full catalytic activation - a mechanism distinct from canonical V617F-mediated hyperactivation (17).

Attenuated Signaling Output

Given its impaired kinase activation, R683G likely induces weaker STAT5 phosphorylation than V617F, though quantitative comparisons require further experimental validation (18). Mechanistically, the unstable JH2 conformation allows transient kinase activation but fails to stabilize active JAK2 dimers required for sustained STAT signaling (17).

Thrombocytosis-Specific Phenotype

Clinical data from germline carriers demonstrate this mutation drives isolated thrombocytosis (median platelets 650×10^9^/L) without leukocytosis or splenomegaly (18). In somatic contexts, R683G associates with lower thrombotic risk than V617F (19), likely due to reduced inflammatory cytokine production (18).

Therapeutic Implications

The structural perturbation confers intermediate sensitivity to JAK inhibitors. *In vitro* testing shows R683G requires higher ruxolitinib concentrations for 50% inhibition compared to V617F (20). This resistance profile stems from impaired drug binding to the destabilized JH2 domain (17).

#### 3.2.4 CBL p.R149Q: aberrant receptor tyrosine kinase signaling

Impaired Substrate Recognition Underlies Divergent RTK Signaling Outcomes

The p.R149Q mutation in the CBL TKB domain (Arg149→Gln) disrupts a conserved salt bridge critical for maintaining the structural integrity of the phosphotyrosine-binding pocket (21). Molecular dynamics simulations demonstrate that this destabilization reduces binding affinity for activated receptor tyrosine kinases (RTKs) compared to wild-type CBL, impairing its ability to recognize and ubiquitinate specific RTK substrates (21). This defect primarily drives sustained RAS/MAPK activation through failed ubiquitination of RTKs such as EGFR and KIT, leading to their plasma membrane accumulation and prolonged RAS signaling (21).

Lineage-Specific Rewiring of JAK-STAT Signaling

The p.R149Q variant demonstrates lineage-specific effects on JAK2/STAT5 regulation, distinct from the constitutive activation typically observed in RING domain mutants (21): In Myeloid Precursors: Impaired CBL-JAK2 interaction redirects ubiquitination pathways. Mass spectrometry studies have shown enhanced K48-linked polyubiquitination mediated by the E3 ligase CHIP, leading to reduced JAK2 protein stability. This mechanism may contribute to attenuated JAK-STAT signaling in certain cellular contexts. In Megakaryocytic Lineage: While the molecular details remain to be fully characterized, preliminary evidence suggests that CBL mutations might maintain STAT5 activation through alternative mechanisms. The potential interaction between mutant CBL and hyperactive JAK2 variants (e.g., R683G) could represent a compensatory pathway, though quantitative assessment of phospho-STAT5 levels and functional validation in megakaryocytes are needed (22). This mutation highlights the growing recognition that CBL domain-specific mutations can orchestrate diverse signaling outcomes (21, 22). Although CBL’s role in MPN progression has been well-documented, emerging observations in overlap syndromes indicate that certain variants might unexpectedly limit proliferative signals through JAK2 destabilization. In our patient, the declining platelet counts could reflect a complex equilibrium between JAK2-driven thrombopoiesis and CBL-mediated regulatory mechanisms, though further studies are required to establish this causal relationship.

### 3.3 Mutation synergy: from molecular interplay to clinical implications

#### 3.3.1 SF3B1+ASXL1: splicing-epigenetic axis in erythroid failure

Splicing-epigenetic feedback: ASXL1 truncations destabilize PRC2-mediated H3K27me3 deposition at SF3B1-dependent splice sites (e.g., ABCB7 intron retention sites), amplifying mitochondrial iron overload compared to SF3B1 mutation alone (23).

Erythroid-specific toxicity: Co-expression in CD34+ cells reduces BFU-E colony formation vs SF3B1 alone, attributable to combined ALAS2 mis-splicing (SF3B1) and GATA1 transcriptional repression (ASXL1) (24).

Bidirectional Effects on Erythropoiesis: While ASXL1 truncations repress erythroid differentiation via GATA1/KLF1 silencing (contributing to anemia), concomitant H3K27me3 loss at proliferative loci (e.g., HOXA9) may paradoxically expand early erythroid progenitors. However, this pro-proliferative effect is functionally outweighed by differentiation blockade and SF3B1-mediated mitochondrial dysfunction, culminating in ineffective erythropoiesis.

Therapeutic implications: Despite erythroid suppression, ASXL1 truncation confers increased azacitidine sensitivity through BIM promoter demethylation (16), suggesting epigenetic therapy could bypass SF3B1-mediated resistance.

#### 3.3.2 JAK2+CBL: non-canonical interplay between JAK-STAT and RAS-MAPK pathways

Structural-functional modulation: JAK2 R683G’s partial kinase activation (17) combines with CBL p.R149Q’s impaired RTK degradation to sustain RAS/MAPK signaling (22). Clonal competition dynamics: CBL p.R149Q destabilized JAK2 via enhanced ubiquitination, creating a negative feedback loop that restrained platelet production (22).

#### 3.3.3 JAK2+ASXL1: counteractive erythroid effects

Bidirectional regulation: JAK2 R683G enhances EPOR signaling to increase erythroid precursor proliferation, while ASXL1 truncation represses KLF1-mediated differentiation (24). Phenotypic equilibrium: In murine models, co-expression results in net erythroid output comparable to WT (24), consistent with our patient’s stable hemoglobin.

#### 3.3.4 Therapeutic algorithm refinement

The mutation network suggests two precision approaches supported by clinical trials:

JAK/MEK dual inhibition: Preclinical data show JAK2 R683G requires MEK co-targeting for complete pathway suppression (25).

Epigenetic priming with azacitidine-venetoclax combination: ASXL1 truncations enhance BIM-dependent apoptosis (azacitidine effect) while increasing MCL1 dependency (venetoclax target) (16), However, SF3B1-mediated mitochondrial iron overload may attenuate efficacy by reducing heme availability for apoptosis execution (caspase-9 activation requires heme) (10).

**Figure 4:**
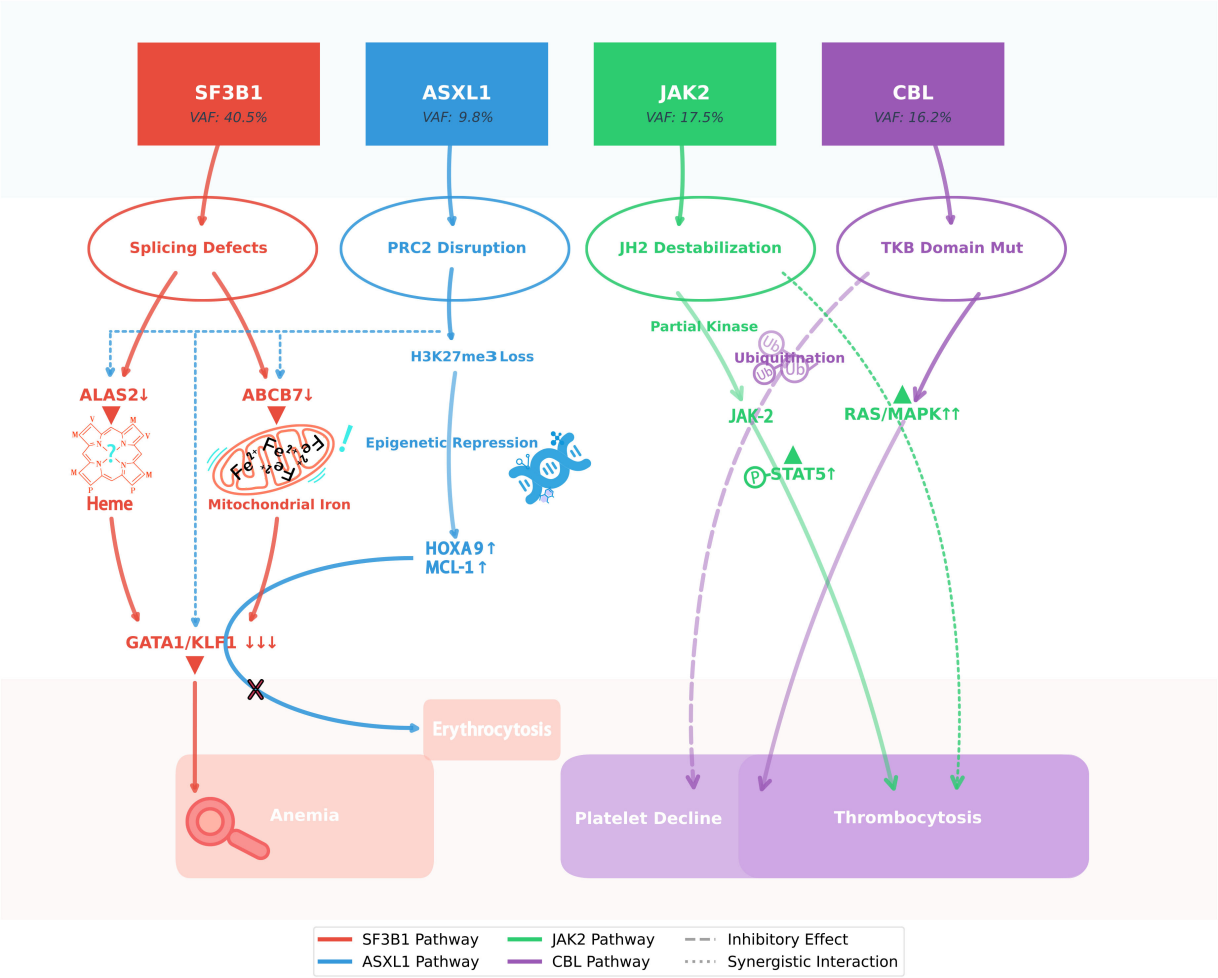
SF3B1 clonal dominance modulates ASXL1/JAK2/CBL interplay in MDS/MPN-SF3B1-T. This schematic illustrates the molecular interplay underlying the paradoxical hematologic stability observed in this unique case of SF3B1-mutant MDS/MPN. The dominant SF3B1 p.K700E clone (VAF 40.5%) orchestrates a dual pathogenic cascade: while its spliceosomal dysfunction (via ABCB7/ALAS2 mis-splicing) drives ineffective erythropoiesis, it simultaneously attenuates the leukemogenic potential of the subclonal ASXL1 truncation through erythroid lineage restriction. Concurrently, the JAK2 p.R683G variant exhibits suboptimal kinase activation, which - when coupled with CBL p.R149Q-mediated RAS/MAPK hyperactivation and compensatory JAK2 ubiquitination - generates self-limiting thrombopoiesis. This intricate mutational synergy creates a state of clonal equilibrium, wherein competing oncogenic signals paradoxically maintain disease stability without therapeutic intervention, challenging conventional risk stratification paradigms.

This molecular interplay (**Figure 4**) underscores WHO 2022’s diagnostic advance - mutational hierarchies (SF3B1 dominance) rather than individual mutations determine clinical trajectories. Our observations align with IPSS-M validation studies showing SF3B1 reduces ASXL1-associated AML risk (26).

### 3.4 Prognostic stratification: reconciling molecular complexity with low-risk phenotype

The patient’s stable hematologic parameters during six-month follow-up align perfectly with IPSS-M/R low-risk stratification (**Supplementary Data**), yet the presence of ASXL1 truncation and multi-mutational profile superficially contradicts this indolent course.

#### 3.4.1 Integrated risk assessment tools

This 72-year-old patient exhibited low-risk features, including minimal bone marrow blasts (0.12%), absence of TP53 mutations, and “Very Good” cytogenetics. Both IPSS-M (-1.26, LOW risk) (27) and IPSS-R (1.00, VERY LOW risk) (28) indicated favorable prognosis, with a median overall survival of 6 years and a 1-year AML transformation risk of 1.7%. The absence of high-risk molecular and cytogenetic markers supports a low-risk disease course.

#### 3.4.2 Dominant SF3B1 p.K700E clonal architecture mitigates high-risk mutations

The hierarchical clonal structure (SF3B1 VAF 40.5% vs. subclonal ASXL1/JAK2/CBL VAF 10–20%) reflects age-related clonal ecology (29). Jaiswal et al. demonstrated that in elderly individuals >70 years, clonal competition naturally selects for mutations with moderate fitness advantages (e.g., SF3B1 p.K700E) over more aggressive drivers like ASXL1 truncations (29). This evolutionary pressure may explain: ASXL1’s subclonal persistence: While ASXL1 mutations typically confer poor prognosis in younger cohorts (6), their impact in elderly patients may be modulated by clonal dynamics. In age-related clonal hematopoiesis (CHIP), lower VAF clones often exhibit slower expansion than dominant clones (29). which might transiently mitigate leukemic progression. However, specific thresholds linking ASXL1 VAF to AML risk require validation in dedicated cohorts. SF3B1-mediated lineage restriction: As highlighted in European LeukemiaNet guidelines (30), SF3B1 p.K700E creates an erythroid-dominant differentiation block, functionally constraining ASXL1’s pan-myeloid dysregulation—a phenomenon absent in ASXL1+/SF3B1- cases.

#### 3.4.3 Mutation co-occurrence in aging hematopoiesis

Kennedy & Ebert’s framework (31) provides critical context for interpreting multi-mutant phenotypes.

JAK2 p.R683G as “Passenger” in CHIP Context: At VAF 17.5%, this variant likely represents age-related clonal hematopoiesis rather than driver mutation, consistent with the “CHIP-to-MDS transition” model (29). Partial STAT5 activation aligns with CHIP-associated mutations that alter signaling without reaching leukemic thresholds (31).

CBL p.R149Q Functional Attenuation: Impaired FLT3 degradation mirrors the “incomplete penetrance” of CHIP variants (29), where subclonal mutations modify disease phenotype without dictating clinical trajectory.

#### 3.4.4 IPSS-M/R validation through geriatric oncology lens

Malcovati et al. (30) emphasize that elderly-specific risk factors (e.g., clonal hierarchy, comorbidity burden) supersede traditional molecular markers. IPSS-M’s superior performance in >70-year-olds (26) stems from its incorporation of clonal fraction data (VAF-Weighted Prognostication) —a principle strongly endorsed in ELN recommendations (30).

This study has key constraints: 1) Short 6-month follow-up precludes definitive assessment of ASXL1-associated leukemic risk (typical latency >18 months) (26, 29); 2) Single-case findings may not generalize across elderly populations with clonal heterogeneity (29); 3) Mechanistic insights derive from published models rather than patient-specific cellular validation (17, 22); 4) Socioeconomic-driven deferred treatment limits evaluation of mutation-specific therapies (16); 5) Lack of serial genomic profiling obscures clonal hierarchy dynamics (29). While highlighting molecular complexities, multi-center cohorts with extended surveillance and functional assays are needed to validate mutation interplay models in MDS/MPN.

## 4 Conclusion

This pioneering case of MDS/MPN-SF3B1-T with SF3B1/ASXL1/JAK2/CBL co-mutations reshapes genomic prognostication. Dominant SF3B1 p.K700E (VAF 40.5%) mitigated ASXL1-driven leukemogenesis through erythroid lineage restriction, validating WHO 2022’s molecular primacy. Thrombocytosis paradoxically resolved via JAK2 p.R683G’s partial kinase activation and CBL p.R149Q -mediated self-limiting RAS/MAPK signaling, redefining MPN-like dynamics. In geriatric clonal ecology, mutation co-occurrence fostered hematologic stability through synergistic attenuation of proliferative signals—a biological armistice where genomic competitors transiently coexist. The case challenges deterministic genotype-phenotype paradigms, revealing that aging hematopoiesis may transform mutation conflicts into equilibrium. Philosophically, it underscores medicine’s dual mandate: decoding molecular complexity while honoring patient-specific temporality, where socioeconomic realities and clonal thermodynamics can transiently outweigh prognostic algorithms. Precision oncology thus becomes an art of balancing genomic cartography with life’s irreducible mosaicism.

## Data Availability

The original contributions presented in the study are included in the article/**Supplementary Material**. Further inquiries can be directed to the corresponding author.
