# Supplementary material for: Case Report: Myelodysplastic/myeloproliferative neoplasm with concurrent SF3B1, ASXL1, JAK2 and CBL mutations and <15% bone marrow ringed sideroblasts
Source: Front Oncol. 2025 Jul 23;15:1622820. doi: 10.3389/fonc.2025.1622820 (PMC12327088; doi:10.3389/fonc.2025.1622820)
Supplement: Supplementary file 1 [file DataSheet1.docx]

Supplementary Material

# Supplementary Figures

1A
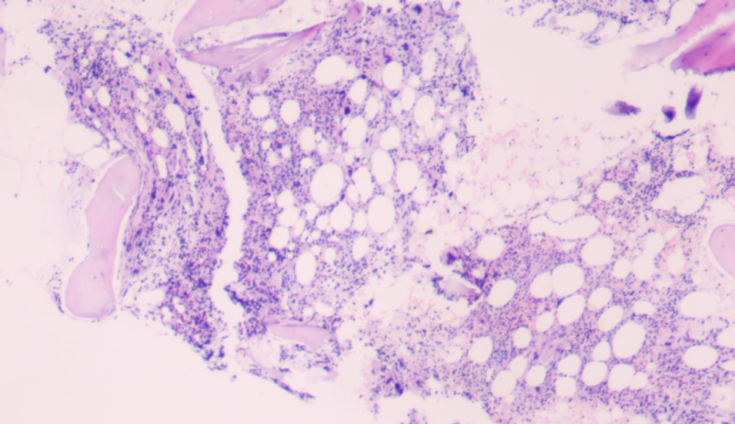


1B
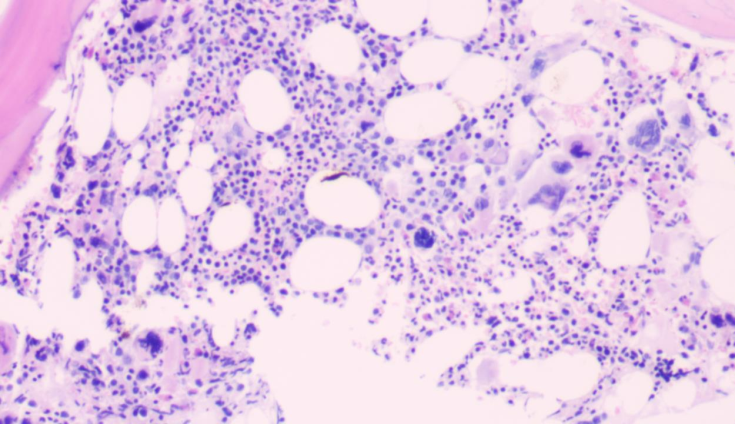


1C
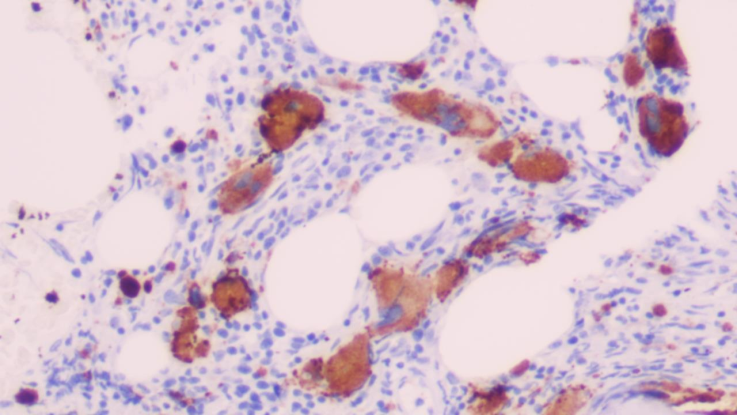


1D
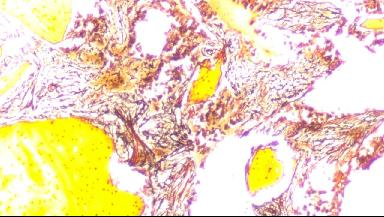


**Supplementary Figures S1A-D**

Bone Marrow Biopsy Report：

Specimen Collection

• Gross examination: One pale-yellow tissue fragment measuring 0.8 × 0.2 × 0.2 cm, with no macroscopic abnormalities.

Microscopic Findings

1. Cellularity: Normocellular marrow (hematopoietic area ~50%) with a normal myeloid-to-erythroid (M:E) ratio.

2. Myeloid series: Predominantly mature granulocytes, with rare scattered immature forms.

3. Erythroid series: Composed mainly of intermediate/late-stage normoblasts.

4. Megakaryocytes: Slightly increased, mostly lobulated nuclei, occasional pyknotic nuclei.

5. Lymphocytes: Scattered, minimal infiltration.

6. Stromal changes: Collagen fibrosis observed in the interstitium.

Ancillary Studies

• Immunohistochemistry (IHC):

• CD34: Small vessels (+), round nuclei (−).

• CD71: Multiple small clusters (+).

• CD61: Megakaryocytes (+), rare monolobated forms.

• Special stains:

• Reticulin stain: Myelofibrosis, grade MF-2 (WHO grading system).

Diagnostic Conclusion(Bone marrow biopsy):

• Erythroid hyperplasia (mildly increased normoblasts).

• No significant increase in blasts (<5%).

• No overt megakaryocytic dysplasia.

• MF-2 myelofibrosis (moderate fibrosis).

2
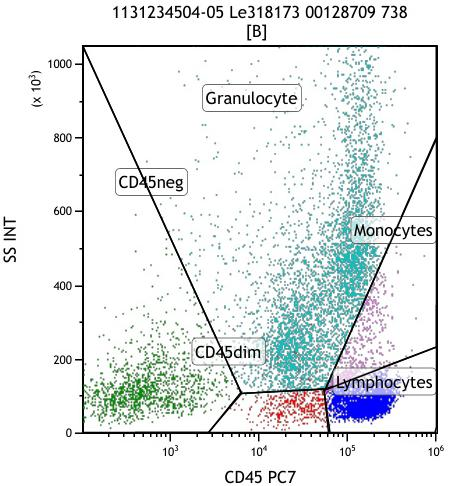


**Supplementary Figure S2**

Flow Cytometry Analysis Report:

Key Findings

1. Blast Population (CD34+CD117+)

• 0.12% of nucleated cells (within normal limits).

• No aberrant immunophenotype detected.

2. Granulocyte Series

• Relative proportion: 55.84% (normal range).

• Immunophenotype: Normal expression of CD13, CD15, CD16, and CD11b (no maturation arrest or dysregulation).

3. Other Cellular Components

• Lymphocytes: 25.88% (normal distribution).

• Monocytes: 4.77% (no significant expansion).

• CD45dim cells: 1.99% (likely erythroid precursors).

• CD45neg cells: 11.52% (consistent with non-hematopoietic elements).

4. No Evidence of High-Risk Clonal Disorders

• No acute leukemia-associated immunophenotype (e.g., aberrant CD7/CD56 co-expression).

• No high-risk MDS-related abnormalities (e.g., loss of CD13/CD16 maturation patterns).

Technical Details

• Total analyzed cells: 7.54 × 10⁶ (adequate for sensitivity).

• Markers evaluated (25 antibodies):

• Myeloid/Progenitor: CD34 (2 fluorochrome conjugates), CD117, HLA-DR, CD33, CD13, CD15, CD16, CD11b, CD64, CD36.

• Erythroid/Megakaryocytic: CD71, CD41.

• Lymphoid: CD19, CD5, CD7, CD2, CD10, CD56.

• Pan-leukocyte: CD45 (5 fluorochrome conjugates).

3A
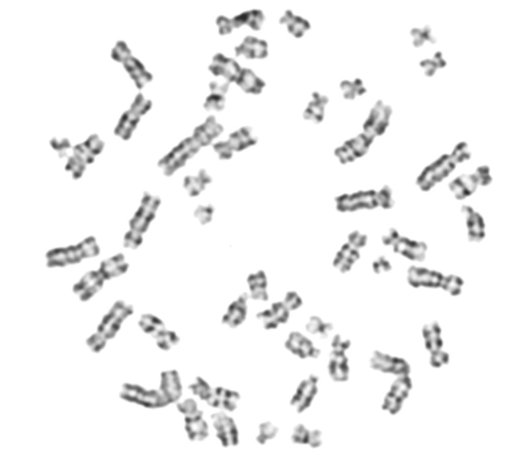


3B
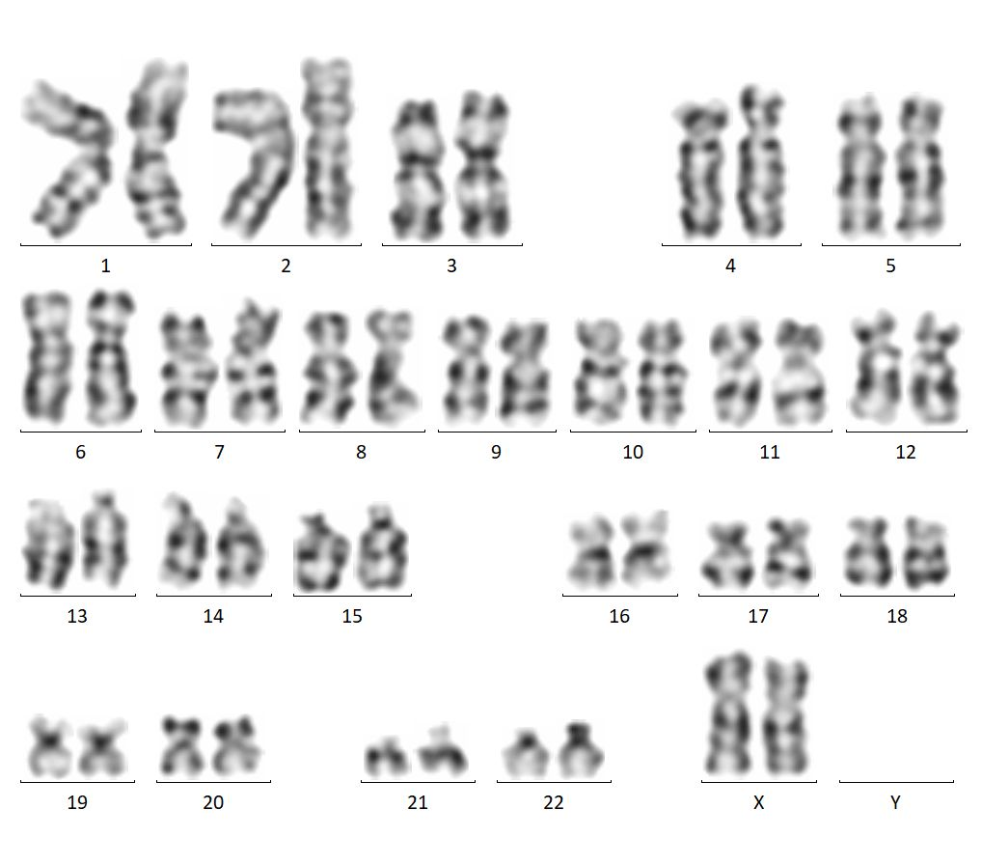


**Supplementary Figures S3A-B**

G-banding analysis of 20 metaphase cells revealed no clonal numerical or structural chromosomal abnormalities.
