# Supplementary material for: Case Report: Myelodysplastic/myeloproliferative neoplasm with concurrent SF3B1, ASXL1, JAK2 and CBL mutations and <15% bone marrow ringed sideroblasts
Source: Front Oncol. 2025 Jul 23;15:1622820. doi: 10.3389/fonc.2025.1622820 (PMC12327088; doi:10.3389/fonc.2025.1622820)
Supplement: Supplementary file 2 [file DataSheet2.pdf]

IPSS-M Risk Calculator Report

Generated on Sunday, May 4, 2025, 9:07 AM

Patient Summary

Bone Marrow Blasts: 0.12 %

Hemoglobin : 9.1 g/dL

Platelet Count: 502 1e9/L

Neutrophil Count: 5.02 1e9/L

Age: 72 years

Cytogenetics Category : Very Good

TP53 Mutation Count: 0

TP53 Maximum VAF: N/A

TP53 locus LOH: No

Mutated Genes: ASXL1, CBL, SF3B1

Missing Genes: 0

Results

IPSS-M Score: -1.26 LOW

IPSS-R Score: 1.00 VERY LOW

IPSS-R Score (Age-adjusted): 1.09 VERY LOW

Endpoints

Leukemia-Free Survival (IPSS-M): 5.9 years (median)

Overall Survival (IPSS-M): 6 years (median)

AML Transformation (IPSS-M): 1.7% (by 1 year)
